# Obtaining consumer perspectives using a citizens’ jury: does the current country of origin labelling in Australia allow for informed food choices?

**DOI:** 10.1186/s12889-016-3900-5

**Published:** 2016-12-09

**Authors:** Elizabeth Withall, Annabelle M. Wilson, Julie Henderson, Emma Tonkin, John Coveney, Samantha B. Meyer, Jacinta Clark, Dean McCullum, Rachel Ankeny, Paul R. Ward

**Affiliations:** 1 Discipline of Public Health, School of Health Sciences, Flinders University, Bedford Park, Adelaide, SA Australia 5042; 2 School of Health Sciences, Flinders University, Bedford Park, Adelaide, SA Australia 5042; 3 School of Public Health and Health Systems, University of Waterloo, 200 University Ave West, Ontario, Canada N2L 3G1; 4 SA Health, Government of South Australia, Adelaide, SA Australia 5000; 5 School of History and Politics, University of Adelaide, Adelaide, SA Australia 5000

**Keywords:** Citizens’ jury, Country of origin, Trust, Food labelling

## Abstract

**Background:**

Contemporary food systems are vast and complex, creating greater distance between consumers and their food. Consequently, consumers are required to put faith in a system of which they have limited knowledge or control. Country of origin labelling (CoOL) is one mechanism that theoretically enables consumer knowledge of provenance of food products. However, this labelling system has recently come under Australian Government review and recommendations for improvements have been proposed. Consumer engagement in this process has been limited. Therefore this study sought to obtain further consumer opinion on the issue of CoOL and to identify the extent to which Australian consumers agree with Australian Government recommendations for improvements.

**Methods:**

A citizens’ jury was conducted with a sample of 14 South Australian consumers to explore their perceptions on whether the CoOL system allows them to make informed food choices, as well as what changes (if any) need to be made to enable informed food choices (recommendations).

**Results:**

Overall, jurors’ perception of usefulness of CoOL, including its ability to enable consumers to make informed food choices, fluctuated throughout the Citizens’ Jury. Initially, the majority of the jurors indicated that the labels allowed informed food choice, however by the end of the session the majority disagreed with this statement. Inconsistencies within jurors’ opinions were observed, particularly following delivery of information from expert witnesses and jury deliberation. Jurors provided recommendations for changes to be made to CoOL, which were similar to those provided in the Australian Government inquiry.

**Conclusions:**

Consumers in this study engaged with the topical issue of CoOL and provided their opinions. Overall, consumers do not think that the current CoOL system in Australia enables consumers to make informed choices. Recommendations for changes, including increasing the size of the label and the label’s font, and standardising its position, were made.

## Background

The globalisation of the food system brings with it greater diversity and choice than ever before. However with it also comes a greater distance and disconnect between consumers and their food. Recent food incidents such as the horsemeat scandal in the UK [1] and melamine in milk in China [2] have caused issues relating to food provenance to become of greater significance for consumers [3]. As a consequence, regulators in many jurisdictions have introduced food traceability requirements. In the EU these are mandatory, while in other jurisdictions such as China and Japan they are voluntary [4]. Bosna and Gebresenbet define food traceability as the capacity “to follow food products throughout the supply chain.” ([4], p.13). They associate traceability with backward and forward follow up of food (tracing and tracking) as well as product history information. Increased food traceability is viewed as increasing consumer satisfaction; improving food safety through enabling food recall and improving the reputation and market access of food which complies with traceability standards [4]. For consumers, food traceability is identified with information about product origin and is related to greater regulation of the food system which in turn, increases trust in both the quality and safety of food [5]. Views of the benefits of food traceability requirements have been found to be culturally specific. German consumers associated food origin with transport and environmental concerns. French and Spanish consumers focus upon the quality of food and Italian consumers associate food origin with food safety [5, 6].

In terms of Australia, first, in March 2014, the Australian Minister for Agriculture and the Minister for Industry requested that the House of Representatives Standing Committee on Agriculture and Industry conduct an inquiry on CoOL and whether the current labelling system provides enough information for consumers to make informed purchasing decisions [7]. Second, the food environment in Australia differs from Europe in that Australian consumers have not experienced food scares of the magnitude experienced in many European countries and as a consequence are generally more trusting of food regulation. Conversely, they distrust food produced overseas, particularly in South East Asian countries which are viewed as not having the same rigour around food regulation [8]. Australia has traditionally produced much of its own produce, with 90% of fresh food produced within Australia in 2011–12. Globalisation and greater consumption of processed foods have contributed to an increase in food importing to Australia leading to greater concerns about the provenance of food [9]. Third, food regulation in Australia occurs over multiple levels of governance [10]. The development of food policy is the responsibility of the Australia and New Zealand Ministerial Forum on Food Regulation which is comprised of the Commonwealth, State, Territory and New Zealand Health Ministers along with Ministers with other relevant portfolios such as primary production [11] while the establishment of food standards is the responsibility of Food Standards Australia New Zealand (FSANZ) a bi-national semi-governmental regulatory agency [12]. CoOL is managed by two regulatory frameworks. The Food Standards Code requires that most packaged and unpackaged foods must have CoOL, while Australian Consumer Law (ACL) provides a series of rules around any claims about a food’s country of origin. CoOL generally identifies where the food is grown, produced or made [7]. The claims of ‘grown in …’ and ‘produced in …’ are premium claims where the significant ingredients and all or virtually all of the processing occur in the claimed country. A third type of claim, ‘made in …’ relates to the manufacture and not the ingredients, and requires that the product was substantially transformed and more than 50% of the costs of manufacture take place in the claimed country. The mere reconstitution of an imported fruit juice concentrate would not satisfy the requirement of this type of claim [13]. The food industry often uses a claim of ‘made in … from local and imported ingredients’. This allows for seasonal or other variability in the country of origin of ingredients – however the product must still satisfy the conditions of being substantially transformed and more that 50% of the costs of manufacture taking place in the claimed country. The enforcement of CoOL is a joint responsibility of those responsible for the enforcement of the Food Standards Code, the states and territories, and the Australian Consumer and Competition Commission (ACCC) and the respective state and territory fair trade commissions [7]. Finally, there is a lack of previous research in Australia about consumer views on country of origin labelling including a lack of information about the views of Australian consumer views on CoOL, in particular in relation to the recommendations made by the Australian Government.

This study sought consumer views of CoOL through deliberative public engagement.

Deliberative public engagement involves the public in discussions about policies and other value-laden issues [14] with the goal of involving them in public decision-making [15]. It involves dialogue between members of the public and sponsors [15] and has been described as a “talk-centric democratic theory” in which the legitimacy of government is associated with public accountability to citizens [16]. As such, deliberative public engagement is viewed as a means of promoting active citizenship [16].

In light of the points outlined above, this study sought to explore the views of Australian consumers about country of origin labelling (CoOL), and in particular changes that could be made to the current system, with a view of comparing these to the recommendations made by the Australian Government during their inquiry. This study is timely and novel as it responds to the House of Representatives Standing Committee on Agriculture and Industry inquiry regarding the extent to which the current labelling system provides enough information for consumers to make informed purchasing decisions [7].

## Methods

### Approach to deliberative engagement

Public participation in deliberative forums is common in public health and health policy research and citizens’ juries are have been reported by authors as a preferred method of deliberative engagement in public health [17, 18]. Citizens’ juries are a technique used for deliberative engagement, based on the legal jury system [19], with a focus on jurors deliberating together to come to an agreed verdict [20]. In this process, lay public participants, also known as “jurors”, take part in 3 phases: (1) knowledge building, where relevant information is presented by expert “witnesses”; (2) cross examination, where the jury is given the opportunity to ask questions of the witnesses to further their understanding of an issue; and (3) deliberation, where jurors, guided by a facilitator, work together to arrive at a verdict, often in the form of an answer to a question or a list of recommendations [21].

Deliberative democracy methods have been shown to be an effective way for researchers to understand public priorities [19, 22, 23], demonstrate a commitment to public participation [21, 22], to educate the public on a particular issue [24, 25] and to offer policymakers the promise of greater transparency and public accountability [17]. Furthermore, citizens’ juries have been praised for the rich and meaningful data they collect [19, 26, 27]. Particularly when public knowledge of an issue is low [28], the jury process educates jurors and exposes them to different points of view and this has been found to deliver quality results when compared to other forms of public participation [29]. This quality of results is attributed to the fact that jurors are given an opportunity to deeply engage with the topic being addressed [30] and have the opportunity to work through the question being asked [31, 32] in a group that is small enough to allow effective deliberation [33]. Members of the public in this study were asked to what extent they agreed with a number of statements about CoOL, and to consider, through a group discussion which was subjected to a qualitative analysis, what changes (if any) they would recommend to enable CoOL to enable informed food choices.

### Recruitment of jurors

Recruitment of jurors used stratified sampling through a market research company. Fifteen participants were drawn from a database of over 40,000 people in Adelaide, South Australia. This recruitment strategy, including the number of participants, has previously been used to recruit jurors for citizens’ juries [19, 34] and has been associated with reduction in bias [33] and the inclusion of underrepresented voices in the jury [30, 35]. The jurors were selected in order to balance a range of demographic factors: age (18 years and over), socio-economic status (via suburb of residence), employment status (employed or unemployed), sex (male or female), political status (Labor, Liberal, Greens or swinging voter) and place of birth (Australia or overseas). These demographic factors had previously been found to impact knowledge of and trust in the food system [36–39] and views of the importance of food traceability [6] and hence were considered to be potentially relevant to jurors’ views about CoOL. Given that we wished to utilise electronic polling as part of the session, it was an eligibility requirement that jurors have mobile phones. This was not considered to introduce selection bias given that approximately 95% of the population aged over 16 years in Australia currently owns a mobile phone [40]. Each juror gave written, informed consent to take part in the Citizens’ Jury, including permission for the deliberation to be audio recorded and transcribed verbatim. Jurors received financial reimbursement to cover the costs of their time and travel to the session as well as any mobile phone costs relating to the polling.

### Recruitment of expert witnesses

Professional contacts from the field of food regulation, economics, public health and law were identified by members of the research team and invited to take part in this project as expert witnesses. The role of witnesses in a citizens’ jury is to inform jurors about evidence from a range of perspectives on the topic under consideration, so that they are able to reach informed decisions and provide recommendations on the topic through deliberation [21].

A total of four witnesses were recruited, which is consistent with the number of expert witnesses typically used in other citizens’ juries [26]. Two witnesses provided evidence in support of, and two evidence against, the current CoOL and its ability to enable consumers to make informed food choices. Each witness was asked which side they would prefer to speak for. The witnesses for retaining current CoOL were a public health scientist and law academic while a public health academic and consumer advocate spoke against the current CoOL. Witnesses received a small gift to thank them for their participation.

### Sitting of the citizens’ jury

The Citizen’s Jury session was a four hour, one-off session conducted in Adelaide, South Australia, in October 2014. One juror did not attend the session; therefore a total of fourteen jurors formed the final jury (see Table 1 for juror characteristics). Traditionally Citizens’ Juries run for several days or weeks depending on the complexity of the issues at hand [30]; however, the issues relating to CoOL were thought to be specific enough to be considered and deliberated effectively in a session of this length. Shorter citizens’ juries have been effective in delivering outcomes (results or findings) successfully [33, 41]. A central disadvantage of a shorter citizens’ jury is allowing sufficient time for deliberation [34].

**Table 1:** Juror characteristics

Juror^a^	Age	Sex (M/F)	Occupation	Australian born (Yes/No)	Political preference (Labor/Liberal)
Bruce	72	M	Aged Pension	No	Swinging
Matthew	58	M	Retired	Yes	Liberal
Michelle	48	F	Legal secretary	No	Labor
Emily	36	F	Student	No	Liberal
Aaron	36	M	Social worker	Yes	Labor
Naomi	18	F	Check out	Yes	Liberal
Lloyd	74	M	Retired Builder	Yes	Swinging
Deborah	59	F	Author	No	Labor
Claire	48	F	Food & beverage attendant	Yes	Liberal
Linda	48	F	Teacher	Yes	Greens
Raj	37	M	Disability Support Worker	No	Labor
Petra	34	F	Marketing Co-ordinator	No	Greens
Andrew	31	M	Electrician	Yes	Liberal
William	19	M	Unemployed	Yes	Swinging

^a^jurors were given pseudonyms

At the beginning of the session, information about citizens’ juries and an outline of the current CoOL requirements of food in Australia was provided by a member of the research team (author removed for blind peer review). Expert witnesses were given 10 min each to present their evidence on the issue and answer questions from jurors. Jurors were then broken into two groups with a facilitator (authors removed for blind peer review) to discuss the topic in greater detail, and the witnesses were available to answer questions that arose. The facilitator’s role was to keep the discussion focussed on the topic and they were briefed about their role prior to facilitating the discussions. The smaller groups were then reunited to report back on their deliberation and reach an overall verdict on the topic. The deliberation sessions were audio recorded.

### Electronic polls

**Fig. 1:**
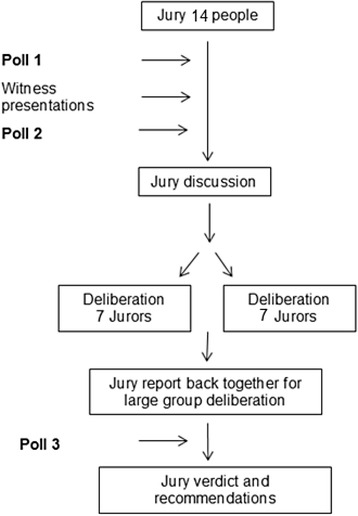
Outline of session

Over the course of the session, jurors were asked to complete a series of electronic polls (refer to Fig. 1). The purpose of these polls was to identify the jurors’ opinions on statements pertaining to the central topic and whether or not these changed over the course of the session. The aim of this part of the study was to be able to identify what discussion topics, if any, appear to sway opinions. The poll statements and response options are outlined in Table 2. The electronic polls were conducted using the Poll Everywhere^1^ audience participation software, which allowed jurors to use their mobile phones to text a code that indicated their response. These results could be instantly collated and displayed to all jurors during the session in anonymised and aggregated form.

**Table 2:** Poll statements and options

	Statement	Options
1	The current country of origin labelling of food allows me to make informed food choices	Agree
		Disagree
		Unsure
		Neutral
2	I trust that country of origin labels are technically correct (i.e., information is accurate)	Agree
		Disagree
		Unsure
		Neutral
3	I trust that country of origin labels are meaningfully correct (i.e., not misleading)	Agree
		Disagree
		Unsure
		Neutral
4	I have confidence in the current written country of origin labelling standards (i.e., what is required to have country of origin labelling and how requirements must be met by companies)	Agree
		Disagree
		Unsure
		Neutral
5	I have confidence that country of origin labelling is currently adequately checked and requirements are enforced	Agree
		Disagree
		Unsure
		Neutral

Some additional background questions were asked of jurors prior to the first poll. Jurors were asked if it is important for them to know where their food comes from; their use of CoOL when food shopping; and their overall trust of food that originates from Australia. These questions were considered to provide important background information, however they were only asked once as they were not likely to change during the course of the session.

### Jury deliberations and qualitative analysis

De-identified transcripts were created from audiotapes made of both of the small group jury deliberations and imported into NVivo 10 (QSR International, Doncaster). Two researchers independently coded the transcripts into key themes (authors removed for blind peer review) using thematic analysis as described by Braun and Clarke [42]. This involved six stages: familiarizing oneself with the data, generating initial codes, searching for themes, reviewing themes, defining and naming themes and producing the report [42]. Initial codes were generated from the transcripts as new themes recurred as analysis progressed.

The codes identified from the two researchers were then compared and analysed for similarity in order to ensure validity. Consistent coding and agreement were found.

## Results

### Poll results

All of the jurors (*n* = 14) completed each of the polls. The background poll identified that knowledge of the country in which food has come from was important for all of the jurors (*n* = 14). The majority (79%, *n* = 11) indicated that they currently use CoOL when food shopping, while others suggested that they do not use them (14%, *n* = 2) or they are not sure whether they use them (7%, *n* = 1).

**Fig. 2:**
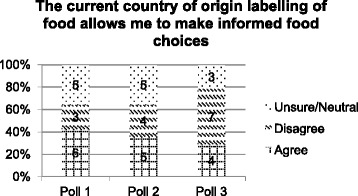
Poll results to statement 1

The central statement considered by the citizens’ jury was statement 1: ‘The current country of origin labelling of food allows me to make informed food choices.’ At poll 1, the majority of jurors agreed (*n* = 6; 43%) with the statement (Fig. 2), similar numbers were also either unsure or neutral (*n* = 5; 36%), and fewer disagreed with the statement (*n* = 3, 21%). Following witness presentations (poll 2), there was a small shift in responses toward more respondents disagreeing (Fig. 2), and this trend continued at poll 3, where half of the jurors disagreed that current CoOL allowed them to make informed food choices (*n* = 7; 50%). Both the number of jurors who were unsure or neutral (*n* = 3; 21%), and the number of jurors who agreed (*n* = 4, 29%) with the statement decreased.

**Fig. 3:**
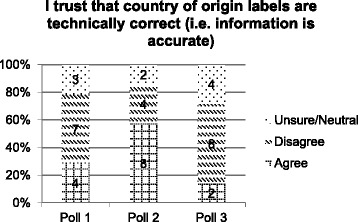
Poll results to statement 2

**Fig. 4:**
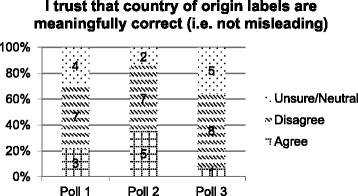
Poll results to statement 3

Statements 2 and 3 consider trust in CoOL in terms of the information being technically correct (i.e., accurate) and meaningfully correct (i.e., not misleading) respectively. At poll 1 the response to these statements was largely similar with 50% (*n* = 7) of jurors disagreeing, (i.e., that the labelling is not technically nor meaningfully correct), and the remainder split between agreement and neutral or unsure responses. At poll 2, following witness presentations, there was an increase in the number of jurors who agreed that CoOL was technically and also meaningfully correct (Figs. 3 and 4). By poll 3, after deliberation the responses to both statements were again similar with the majority disagreeing (*n* = 8, 57% - both statements 2 and 3), and growth in the number of jurors who were unsure or neutral, and decreases in the number who agreed with the statements.

Confidence in the written standard increased at poll 2 (Statement 4; see Table 2), whilst confidence in the policing of CoOL and enforcement of requirements decreased.

The poll results identify fluctuations within responses across the three time points of the session. Transcripts from the deliberations are explored herein in greater detail to assist in the interpretation of the poll results and identification of when and why these fluctuations occurred.

### Jury deliberations and recommendations

The discussion that follows identifies juror perceptions of the current labelling process, considerations about changing the labelling process and recommendations for changes to labelling, identified through the deliberative discussion process.

### Perceptions of current labelling practices

#### No concerns with current CoOL

As indicated above; a small proportion of jurors (*n* = 5) did not have concerns with current CoOL. Reasons cited included jurors already held trust in the food system, prioritising other interests and CoOL increases the complexity of shopping.

#### Trust in the food system

For these jurors, trust in the food system was the most important way to ensure food was safe, and therefore CoOL was less relevant. Matthew stated for example, that “*I do have a trust here that there’s enough checks and balances, that it’s okay*.” A similar view was expressed by Emily who, while preferring to buy Australian food, noted that:

*I don’t always have time to look for it [country of origin labelling], I tend to just trust. I have to trust that they’re, you know, putting the right measures in place to make sure we’re getting good quality food*. - Emily

These jurors cited trust in quarantine practices, supermarkets and the interests of the food industry in ensuring that their food is safe to consume and of ‘good quality’. The citizen’s jury process however led other jurors to question their trust in the food system. For example, Naomi stated:

*… I do the shopping for mum because she just doesn’t have enough time at all and to be honest I don’t even look for any of this stuff. I have complete trust in the system and watching all this tonight has made me question…*(emphasis added). - Naomi

#### Prioritising other interests

For other jurors, prioritising other interests meant they had limited concerns about CoOL. For example Emily said:

*…if I walk into Coles or Woollies [the two major supermarket retailers in Australia] to be very honest for me in terms of labelling I look more into the nutrition part as opposed to the country of origin. That, to me – because at the end of the day I’m a mum with three kids so sugar’s sugar, milk is milk. Home brand [generic foodstuffs] is still fine with me and I know that my kids are – you know it’s from Australia and it’s fine with me. -* Emily

Implicit within this statement is trust that food sold in Australia regardless of where it is grown, manufactured or packaged – is safe.

#### Complexity of shopping

For other jurors, CoOL added to the complexity of food shopping. For example Claire explained:

*I find that people tend to complicate a lot of things as they go along and I find that why complicate it? People actually want to know about so many things. Like you’ve already got people arguing that nutrition labels have to be specific, so you’ve got that type of people trying to work on getting more tedious information there and you could have this set of people trying to put more information of country of origin. -* Claire

#### Concerns with country of origin labelling

The majority of jurors (*n* = 9), however, identified concerns with CoOL labelling, which centred on three issues: the transparency of current labelling practices; (lack of) education about how to interpret the labels; and the enforcement of CoOL. The confusion associated with labelling was related to the wording of the labels. For one juror labelling was viewed as “*confusing. It’s tricky… now to know what each word means*.” - Michelle

#### Transparency

Particular confusion was noted relating to the difference between ‘made in Australia’ and ‘product of Australia’: “*It could be – the ingredients could have come from elsewhere but as long as it was made here then it gets labelled ‘made in Australia’*.” – Raj

For some jurors (*n* = 5), confusion arises from the use of other types of labels. A male juror highlighted the impact of the ‘Australian made’ label on public perceptions:

*…if people don’t read that [the mandated country of origin labelling]and they just see the green triangle, if they didn’t have that green triangle* 2 *for a product of Australia or made in Australia so it’s to – for people to see that and think ‘oh it’s Australian’. –* Andrew

Another juror cited the use of other Australian-themed symbols as potentially misleading:

*…I swear that some packaging features like …we said with the Koala or the Southern Cross and then it says ‘made in Australia’. Yes, the ‘from local and imported ingredients’ might appear somewhere else but I think people just take it at face value. –* Petra

A third issue in relation to transparency was the size and positioning of the CoOL. Several jurors noted how difficult it typically was to even find the label relating to country of origin. One juror argued that while “*made in Australia’ may appear in quite big lettering*” ‘from imported ingredients’ appears “in *tiny, tiny print somewhere*.” – Linda

#### Education

For other jurors (*n* = 3), the problem is not the labelling but public understanding of what these labels mean: *Maybe the regulation is fine. Like if you want to look you will find it somewhere but if you don’t know how to interpret it, it’s a problem and it’s a problem if you don’t know that you have to look for that, so that’s an educational problem.* – Petra

This juror noted people from developed countries like Australia should have sufficient education to make informed decisions and to ask the right questions. However, this view was challenged by another participant who said *“I was thinking because the normal person and that, you know, are going to the supermarket and I look at the label and I can’t understand half of it because it’s initials, or whatever…”* - Deborah

Participation in the Citizens’ Jury was viewed as promoting consumer education. A third juror said “*we [now] can make an informed choice because we’ve been discussing it but can everybody? Does everyone understand?” -* Andrew

#### Enforcement

A final set of concerns related to the adequacy of enforcement of CoOL. As noted above, the responsibility for enforcement of CoOL is distributed among a number of state, territory and federal agencies, where issues relating to misleading or deceptive conduct are enforced through the ACCC or the respective state/territory counterpart (Parliament of Australia, 2014). Enforcement actions may be initiated following surveillance activities or through public complaints highlighting issues of concern. One juror described the ACCC as “*a toothless tiger.....in that sense. It doesn’t have a huge mandate; it can’t aggressively go after things. It’s got to have stuff brought to its attention*.” – Aaron

In light of this, another juror questioned whether “*people might put something on their label and just hope that no-one finds out*?” - Petra

One juror mentioned a concern related to a perceived loophole with food imported from New Zealand. She stated “*my understanding is that they have things that come in from China but are packaged and processed in some way in New Zealand and can then be labelled as made in New Zealand.*” – Linda

#### Considerations in changing current labelling practices

Despite reservations about current labelling practices, jurors identified three issues to be considered in revising the current standards. The first concern was that revising labelling standards would lead to greater complexity: “*instead of adding more information maybe they should just clarify the information that they have, make it more simpler [sic]*.” – Naomi

For another juror “*the more complex an item is the more you can’t make a rule because there’s so many variants that come in*”. – Matthew

A second consideration is whether changing the CoOL would address problems arising from the enforcement of the labelling.

A third consideration related to cost both to small businesses but also to the consumer. Some jurors argued that the cost of relabelling products may be prohibitive for small businesses while others argued that it would be relative and hence unproblematic:

*A small company may have one or two products; their turnover is whatever. It’s not going to be $60,000 to turn that – change the labelling. It’s going to be relative but it’s only going to be a small proportion of what they’re turning over-* Lloyd

One juror suggested that the cost of relabelling would be passed onto the consumer: “*[r]aise the price by two cents, wouldn’t that cover the cost*?” – Claire

Another juror cited a large Australian company’s unsuccessful experiment with producing Australian products, noting that Australians were not prepared to pay 35 cents extra for Australian made peanut paste.

#### Recommendations of changes to labelling

The changes suggested by the jurors focused upon the transparency of labels and providing more education, and are summarised in Table 3. Notably, the group was unable to reach consensus on which particular changes to CoOL should be prioritised, so the table provides a summary of the range of suggestions for each issue, including those which had overall agreement from the jury.

**Table 3:** Summary of recommended changes to country of food origin labelling

Issue	Suggested improvements
Wording of the label	• Do not add further information but increase clarity of existing information
	• Clarify difference between ‘Product of Australia’ and ‘Made in Australia’
	• Indicate the percentage of the product which is Australian
	• Provide a star rating according to the percentage of Australian product contained
Size	• Increase the size of the label
	• Increase the size of the font
	• Make the font size uniform for all information.
Position of label	• Standardise the position of the CoOL
	• Place the CoOL under the nutrition information panel
Education	• Teach people how to interpret the information on food labels
Deceptive labelling	• Prohibit the use of symbols/images which suggest the product is Australian

## Discussion

This study utilised a citizens’ jury methodology to explore consumer perceptions of the current CoOL system in Australia. Specifically, this study investigated the views of Australian consumers about country of origin labelling (CoOL), and in particular changes that could be made to the current system, with a view of comparing these to the recommendations made by the Australian Government during their inquiry. At poll 3, the majority of jurors disagreed that CoOL enables informed food choices. Although this was not a unanimous verdict, there was less uncertainty and ambivalence than was identified within the first two polls. Overall the polls suggest that juror opinions fluctuated in response to expert information and engaging in deliberation with others; however, the within-poll responses did not always appear to be consistent at first glance. For example, following the witness presentations more jurors disagreed with the statement that CoOL allow for informed food choices; however within the same poll, trust that these labels are technically and meaningfully correct increased. However, given the jurors’ focus on the need for more education about labelling, these initially inconsistent results are reconcilable if interpreted as meaning that although CoOL is technically and meaningfully correct, it still does not promote informed food choices because consumers have not been educated about the technicalities and meanings associated with CoOL, or find it difficult to utilise the labels (given the small font size and variable location). Therefore, it is evident that consumers in this Citizens’ Jury believe that more than a technically and meaningfully correct food label is required to enable a consumer to make an informed food choice.

The jury deliberations and recommendations provide context for understanding jury poll responses. Our data indicate that a minority of jurors were able to influence the majority to change their poll response during deliberation, as there was a large swing in juror opinion between polls 2 (after witness presentations) and 3 (after deliberation). One interesting result was decreasing trust in current labelling practices at poll 3. Following Biltgard [43], if trust is viewed as habitual and unreflexive we could argue that the process of participation in the Citizens’ Jury has undermined trust in the food system. If alternatively, we regard placing trust as involving a conscious act, the receipt of information can presumably reduce uncertainty through increasing capacity to make informed choices. Both views were expressed during the deliberation process with respondents noting that the Citizens’ Jury had made them question previously taken-for-granted assumptions about the food system but had also enabled informed choice. Given that the majority of participants (*n* = 8) had pre-existing concerns or uncertainties about food labelling, the benefits of the process may outweigh impact on trust.

There was however, a small group of participants who maintained trust in food regulation throughout the Citizens’ Jury. Comments from the deliberation process suggest that this may be an issue of concern about other priorities rather than simply trust. Ward et al. [44] found that when consumers receive conflicting information about the food system or feel overloaded by information they may choose to ignore that information in favour for a ‘common sense’ understanding of what is safe. When this occurs trust becomes a default position that allows consumers to manage uncertainty. This approach was also reflected in concerns that revision of CoOL labelling may make the information more complex.

The concerns that were identified by the jurors regarding CoOL included transparency (i.e., language used), the need for consumer education to enable understanding of the labels, more active enforcement of labels and concern about loopholes. In relation to loopholes, all food imported into Australia, including from New Zealand, must have CoOL. In the case of imports from New Zealand under a bi-lateral agreement,^3^ food must comply with New Zealand legal requirements. While mandatory CoOL is not required in New Zealand, where it is provided, as in the case for food exported to Australia, it must not be misleading or deceptive. Merely repackaging an imported product would not satisfy the New Zealand requirements (Parliament of Australia 2014). However the complexity of the CoOL regime, and indeed the complexity of food processing, creates what to consumers are serious loopholes in the regime. The reservations about checking and enforcement of CoOL standards were echoed in the report from the House of Representative Standing Committee on Agriculture and Industry into CoOL (released after the Citizens’ Jury), with noticeable similarities between the recommendations produced from the report and from this citizens’ jury. Specifically, the Committee recommended that the CoOL standards be amended such that the country of origin text increase in size compared with the standard text on the food label. Many of the jurors supported the font size increase to enable the country of origin to be more easily identified on a food label, but they also went further to recommend that the CoOL be in a standardised location on the label.

The jurors were in agreement for consumers to receive education to enable them to interpret labels, although they did not provide any concrete strategies. Similarly, the Committee report described the need for the Australian Government, industry and consumer advocacy groups to develop and implement an education program. The jurors also agreed to recommended prohibition of symbols and images which imply that a product is Australian, similar to recommendation 3 of the Committee report suggesting increased scrutiny of products with mostly or all imported ingredients that use misleading imagery. Furthermore, jurors agreed to recommendations that there should be a means to clarify the difference between ‘Made in Australia’ and ‘Product of Australia’, although were not in agreement regarding how this should be practically implemented (Table 3). The Committee recommended that the Australian Government implement a series of safe harbours for CoOL, for example: ‘Made in [country] from mostly local ingredients’ which would indicate that a product contains more than 50% Australian content. The Committee also recommended that a visual descriptor for this recommendation that represents the safe harbour thresholds of Australian ingredients be included in the contents of a product. Therefore it would appear that the jury provided recommendations that included similar elements to that of the Committee. The consistency between the two sets of recommendations bodes well for public support of any resultant changes to labelling [25, 45].

The finding that the majority of jurors trust Australian food is consistent with previous research [36]. For example, Henderson et al. [8] identified that Australian jurors perceived Australian foods to be safe, despite having limited knowledge about the regulatory mechanisms or processes involved. The trust in local, Australian food from jurors in the current study was so strong that many times deliberations diverted to discussions of imported foods in general and whether or not they are safe. Occasionally this resulted in confusion with jurors then making a connection between seeing imported foods from countries in which they have little trust as a sign that CoOL standards are ‘failing’. However, the fact that they were able to identify imported food demonstrates that CoOL enabled them to make informed choices.

### Limitations

This study is not without its limitations. Firstly, as with the case with many citizens’ juries, it includes a small sample size [21]. While only 14 people participated, Rowe and Frewer note that larger samples may result in non-participation in the deliberation process particularly in shorter citizen’s juries [15]. As a consequence, caution should be taken in generalising results to the wider population. Notwithstanding this, the Citizens’ Jury did provide rich and meaningful data [26] as jurors with a range of demographic characteristics and varied perspectives participated. The duration of the session was relatively short in comparison to other juries in that it was limited to one evening. Although it has been identified that shorter juries can yield similar outcomes to extended juries [33, 41], the authors acknowledge that a longer jury or follow-up session (resources permitting) could have been beneficial.

## Conclusions

The interactive process of the citizens’ jury enabled jurors’ concerns over the current CoOL to be explored. Jurors concluded that current CoOL in Australia does not allow consumers to make informed food choices. The jurors produced a series of recommendations that were similar to those produced by the inquiry conducted by the Australian Government, despite the relatively short timeframe of the Citizens’ Jury session. This is positive, suggesting that the recommendations made by the Government are likely to be supported by consumers. However, given the small sample size of the Citizen’s Jury, more research is needed to confirm and support these findings. To the authors’ knowledge, this study is the first to utilise a citizens’ jury approach to engage consumers in the topical issue of whether the current CoOL allows consumers to make informed food choices, and to compare consumer opinion to recommendations resulting from an Australian Government inquiry. Citizens’ juries as a means of public engagement should be considered for future policy planning and development, especially when it directly affects the public, such as changes to CoOL.

## Abbreviations

ACCC: Australian competition and consumer commission
CoOL: Country of origin labelling
FSANZ: Food standards Australia new Zealand

## Footnotes

1 Poll Everywhere can be accessed via www.polleverywhere.com

2 The ‘green triangle’ noted likely refers to the green and gold triangular kangaroo logo. This is a certified trademark (CTM) to reflect products that are grown and/or made in Australia. Producers apply to use the CTM logo and its use is governed by a series of rules. The CTM can apply to many products, not just food.

3 The Trans-Tasman Mutual Recognition Arrangement is an arrangement between the Commonwealth, State and Territory Governments of Australia and the Government of New Zealand seeking to develop an integrated trans-Tasman economy.

## References

[1] Horse Meat Investigation. [https://www.food.gov.uk/enforcement/monitoring/horse-meat]. Accessed 15 Feb 2014.

[2] Expert meeting to review toxicological aspects of melamine and cyanuric acid. [http://www.who.int/foodsafety/fs_management/Exec_Summary_melamine.pdf]. Accessed 15 Feb 2014.

[3] Meyer SB, Coveney J, Henderson J, Ward PR, Taylor AW (2012). Reconnecting Australian consumers and producers: Identifying problems of distrust. Food Policy.

[4] Bosona T, Gebresenbet G (2013). Food traceability as an integral part of logistics management in food and agriculature supply chain. Food Control.

[5] Van Rijswijk W, Frewer LJ, Menozzi D, Faioli G (2008). Consumer perceptions of tracebility: a cross national comparison of associated benefits. Food Qual Prefer.

[6] Menozzi D, Halawany-Darson R, Mora C, Giraud G (2015). Motives towards traceable food choice: a comparison between French and Italian consumers. Food Control.

[7] Parliament of Australia (2014). Inquiry into country of origin food labelling.

[8] Henderson J, Ward PR, Coveney J, Meyer S (2012). Trust in the Australian food supply: innocent until proven guilty. Health Risk Soc.

[9] Department of Agriculture Fisheries and Forestry [DAFF] (2013). Australian food statistics 2011–2012.

[10] Healy M, Brooke-Taylor S, Liehne P (2003). Reform of food regulation in Australia and New Zealand. Food Control.

[11] The Australia and New Zealand ministerial forum on food regulation. [http://www.health.gov.au/internet/main/publishing.nsf/Content/foodsecretariat-anz.htm]. Accessed 8 Jan 2014.

[12] Winger R (2003). Australia and New Zealand food standards code. Food Control.

[13] Australian Competition and Comsumers Commission (2014). Country of origin claims and the Australian consumer law: a guide for business.

[14] Abelson J, Forest P, Eyles J, Smith P, Martin E, Gauvin P (2003). Deliberations about deliberative methods: Issues in the design and evaluation of public participation processes. Soc Sci Med.

[15] Rowe G, Frewer LJ (2005). A typology of public engagement mechanisms. Sci Technol Hum Values.

[16] Delli Carpini M, Lomax Cook F, Jacobs L (2004). Public deliberation, discursive participation and citizen engagement: a review of empirical literature. Ann Rev Polit Sci.

[17] Degeling C, Carter SM, Rychetnik L (2015). Which public and why deliberate? e A scoping review of public deliberation in public health and health policy research. Soc Sci Med.

[18] Gregory J, Hartz-Karp J, Watson R (2008). Using deliberative techniques to engage the community in policy development. Aust New Zealand J Health Policy.

[19] Elwood P, Longley M (2010). My health: whose responsibility? A jury decides. J Epidemiol Community Health.

[20] Glasner P, Dunkerley D (1999). The new genetics, public involvement, and citizens’ juries: a welsh case study. Health Risk Soc.

[21] Bennett P, Smith SJ (2007). Genetics, insurance and participation: how a citizens’ jury reached its verdict. Soc Sci Med.

[22] Dietrich H, Schibeci R (2003). Beyond public perceptions of gene technology: community participation in public policy in Australia. Public Underst Sci.

[23] Pickard S (1998). Citizenship and consumerism in health care: a critique of citizens’ juries. Soc Policy Adm.

[24] Thornton H (2001). Information and involvement. Health Expect.

[25] Tenbensel T (2002). Interpreting public input into priority-setting: the role of mediating institutions. Health Policy.

[26] Arrighi E, Blancafort S, Jovell AJ, Navarro Rubio MD. Quality of cancer care in Spain: recommendations of a patients’ jury. Eur J Cancer Care. 2014;24:387–94. 10.1111/ecc.12208 24841164

[27] Jones M, Einsiedel E (2011). Institutional policy learning and public consultation: the Canadian xenotransplantation experience. Soc Sci Med.

[28] Rychetnik L, Carter SM, Abelson J, Thornton H, Barratt A, Entwistle VA, Mackenzie G, Salkeld G, Glasziou P (2013). Enhancing citizen engagement in cancer screening through deliberative democracy. J Natl Cancer Inst.

[29] Paul C, Nicholls R, Priest P, McGee R (2008). Making policy decisions about population screening for breast cancer: the role of citizens’ deliberation. Health Policy.

[30] Gooberman-Hill R, Horwood J, Calnan M (2008). Citizens’ juries in planning research priorities: process, engagement and outcome. Health Expect.

[31] Murphy NJ (2005). Citizen deliberation in setting health-care priorities. Health Expect.

[32] Menon D, Stafinski T (2008). Engaging the public in priority-setting for health technology assessment: findings from a citizens’ jury. Health Expect.

[33] Street J, Duszynski K, Krawczyk S, Braunack-Mayer A (2014). The use of citizens’ juries in health policy decision-making: a systematic review. Soc Sci Med.

[34] Henderson J, House E, Coveney J, Meyer S, Ankeny R, Ward P, Calnan M (2013). Evaluating the use of citizens’ juries in food policy: a case study of food regulation. BMC Public Health.

[35] Iredale R, Longley M (2007). From passive subject to active agent: the potential of citizen’s juries for nursing research. Nurse Educ Today.

[36] Holmberg L, Coveney J, Henderson J, Meyer S (2010). What should primary health care practitioners know about factors influencing young people’s food choices?. Australas Med J.

[37] Henderson J, Coveney J, Ward PR, Taylor AW (2011). Farmers are the most trusted part of the Australian food chain: results from a national survey of consumers. Aust N Z J Public Health.

[38] Poppe C, Kjaernes U (2003). Trust in Food in Europe.

[39] Kornelis K, de Jonge J, Frewer L, Dagevos H (2007). Consumer selection of food-safety information sources. Risk Anal.

[40] Neilsen (2014). Australian connected consumer report.

[41] Timotijevic L, Raats MM (2007). Evaluation of two methods of deliberative participation of older people in food-policy development. Health Policy.

[42] Braun V, Clarke V (2006). Using thematic analysis in psychology. Qual Res Psychol.

[43] Bildtgard T (2008). Trust in food in modern and late-modern societies. Soc Sci Inf.

[44] Ward PR, Henderson J, Coveney J, Meyer S (2012). How do South Australian consumers negotiate and respond to information in the media about food and nutrition? The importance of risk, trust and uncertainty. J Sociol.

[45] Kashefi E, Mort M (2004). Grounded citizens’ juries: a tool for health activism?. Health Expect.

